# Sex Differences in the Treatment of Uveal Melanoma in a Group of 1336 Patients

**DOI:** 10.3390/jpm13020353

**Published:** 2023-02-17

**Authors:** Bożena Romanowska-Dixon, Magdalena Dębicka-Kumela, Janusz Śmigielski, Michał Szymon Nowak

**Affiliations:** 1 Department of Ophthalmology and Ophthalmic Oncology, Jagiellonian University Collegium Medicum, 38 Kopernika Str., 31-501 Krakow, Poland; 2 Department of Statistics, State University of Applied Science in Konin, 1 Przyjazni Str., 65-510 Konin, Poland; 3 Institute of Optics and Optometry, University of Social Science, 121 Gdanska Str., 90-519 Lodz, Poland; 4 Provisus Eye Clinic, 112 Redzinska Str., 42-209 Czestochowa, Poland

**Keywords:** uveal melanoma, brachytherapy, proton therapy, enucleation

## Abstract

(1) Background: The aim of this study was to analyze the sex differences in the treatment of uveal melanoma (UM) in a group of 1336 patients from a national referral center during the period 2018–2021. (2) Materials and Methods: The study was designed in a retrospective manner. A total of 1336 patients who were newly diagnosed with UM at the Department of Ophthalmology and Ophthalmic Oncology, Jagiellonian University Collegium Medicum, Krakow, Poland, between 1 January 2018 and 31 December 2021, were included in the study. The demographic and clinical data were compiled, including the sex of patients and the treatment methods. (3) Results: In total, 1336 patients with ocular melanoma were identified, including 726 women (54.34%) and 610 men (45.66%). A total of 49.70% of tumors were localized in the right eye and 50.30% in the left eye. UMs were localized statistically significantly more frequently posterior to the equator of the eye globe in men than in women (79.67% vs. 74.10%, Chi^2 Pearson test *p* = 0.035). Tumors tended to be larger in men, but this difference was not clinically significant. Men were enucleated more often than women (23.44% vs. 18.04%, Chi^2 Pearson test *p* = 0.015). (4) Conclusions: Statistically significant sex differences were found in the treatment of uveal melanoma in a national referral center in Poland, with men being enucleated more often than women.

## 1. Introduction

Uveal melanoma is the most common primary intraocular cancer in adults, with a distinctively different etiology from that of cutaneous melanoma. However, the incidence of uveal melanoma varies across ethnicities and regions worldwide; a north-to-south decreasing gradient in uveal melanoma incidence is observed in Europe. This may be related to the protective effect of ocular pigmentation in southern populations with respect to higher exposure to ultraviolet light at lower latitudes [1,2,3,4,5].

The results of previously published studies revealed sex differences in the incidence of uveal melanoma, but their results were not consistent. Most of them showed an increased age-adjusted incidence rate of ocular melanoma among men [6,7,8,9,10,11,12], but in other large cohort clinical studies with no age adjustment, no sex-based differences were reported [3]. In contrast to those studies, the incidence rate of ocular melanoma in Poland was higher in women [13]; this was attributable to the excess male death rate characteristic to Eastern European countries, which continues to have an effect in Poland [14,15,16]. The other sex differences observed among patients with uveal melanoma included tumor size, localization of the tumor, as well as the development of metastases and mortality rate. In men, tumors tended to be larger and more posterior than in women. Men suffered more metastases, and their mortality rate was also higher than in women (in studies with less than 10 years of follow-up) [13,17,18,19,20].

The treatment of ocular melanoma depends on the tumor localization, size, local extension, visual acuity at presentation, and systemic status. Most patients with posterior tumors are currently treated with plaque brachytherapy. Others are treated with laser photocoagulation, transpupillary thermotherapy, particle beam radiotherapy, gamma knife radiosurgery, local surgical resection, and/or enucleation. The current standard for anterior tumors management is surgical treatment with adjuvant therapy, including brachytherapy. The goal of uveal melanoma treatment is to save the life of the patient; the secondary aims are, in order of priority, to preserve the eye, vision, and cosmetic wishes of the patient [1,3,5,21,22].

The present study aimed to analyze the sex differences in the treatment of uveal melanoma in a group of 1336 patients from a national referral center in Poland in the period 2018–2021. To the best of our knowledge, this is the first study searching for sex differences in the treatment of uveal melanoma.

## 2. Materials and Methods

### 2.1. Data Sources, Patients, and Definitions

The study design was a retrospective case series. All patients who were newly diagnosed with uveal melanoma and treated at a national referral center (Department of Ophthalmology and Ophthalmic Oncology, Jagiellonian University Collegium Medicum, Krakow, Poland) between 1 January 2018, and 31 December 2021, were extracted from the hospital database and included in the study. This hospital database provides medical data, which include the diagnoses coded according to the International Classification of Diseases, 10th Revision (ICD-10); the 3rd edition of the International Classification of Diseases for Oncology (ICD-O-3) codes; and all performed procedures coded using the International Classification of Diseases, 9th Revision (ICD-9) procedure codes and unique National Health Fund (NHF) codes, corresponding to certain hospital procedures. The hospital database also provides demographical features such as the date of birth and sex of patients. The Department of Ophthalmology and Ophthalmic Oncology, Jagiellonian University Collegium Medicum, Krakow, is the referral center for ophthalmic oncology for adults in Poland. The following data were compiled: the sex of patients and their age at the time of diagnosis; the year of diagnosis; clinical findings such as the laterality of the tumor (right or left eye); intra-ocular localization and cancer stage, according to the TNM classification of malignant tumors (both at the time of diagnosis); and applied treatments including plaque radiotherapy (brachytherapy with iodine-125 or rhutenium-106), proton beam irradiation (PBI), local surgery, and/or enucleation of the eye globe. Tumors were clinically classified as localized in the iris, ciliary body, choroid, or mixed (according to their appearance).

### 2.2. Statistical Analyses

All data were entered into the Microsoft Excel database, and commercially available software STATISTICA v. 13.0 PL (StatSoft Polska, Krakow, Poland) was used to perform all statistical analyses. The statistical analyses included the annual incidence analysis of uveal melanoma cases during the period 2018–2021 in the national referral center in Poland (the prevalence rates per 100 women and men were calculated), and analyses of tumor laterality, localizations of tumors, cancer stages, and treatment methods (the sex distribution was explored by the Chi squared (χ^2^) test). The treatment methods were also analyzed according to anterior or posterior localization (to the equator of the eye) at the time of melanoma diagnosis. Then, univariate and multivariate logistic regression models were constructed to investigate the association between the medical management of uveal melanoma (globe-sparing therapies vs. enucleation) and patients’ sex, the localization of melanomas (anterior vs. posterior to the equator of the eye), and sizes, according to the TNM classification. *p*-values of less than 0.05 were considered statistically significant.

This study adhered to the tenets of the Declaration of Helsinki for research involving human subjects, and the study protocol was approved by the Institutional Review Board of the Jagiellonian University Collegium Medicum (informed consent was waived).

## 3. Results

**Figure 1:**
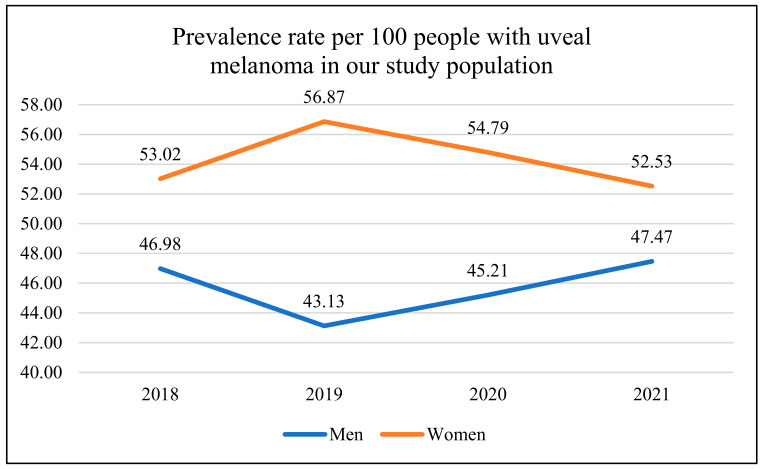
Sex differences in prevalence rate per 100 people included in the present study (with uveal melanoma) in the national referral center in Poland between 2018 and 2021.

The mean age at the time of diagnosis was 63.84 ± 13.86 years, and this was slightly higher among women (64.26 ± 14.33 years) than men (63.24 ± 13.27 years). In total, 1336 patients with ocular melanoma were identified in the national referral center in Poland between January 1, 2018, and December 31, 2021, including 726 women (54.34%) and 610 men (45.66%) (Table 1). The sex distribution in our study population was similar to that found among patients with ocular melanoma in the whole population of Poland (statistical analysis—Chi squared test: *χ2* = 0.20, *p* = 0.6570) [13]. The prevalence rate per 100 people varied from 52.53 in 2021 to 56.87 in 2019 in women, and from 43.13 in 2019 to 47,47 in 2021 in men, respectively (Figure 1). In total, 49.70% of tumors were localized in the right eye and 50.30% in the left eye. In men, 51.15% of UMs were found in the right eye, while in women 51.52% were found in the left eye; however, this difference was not statistically significant. In our study population, the uveal melanomas were localized statistically significantly more frequently posterior to the equator of the eye globe in men than in women (79.67% vs. 74.10%, *p* = 0.035) (Table 2). At the time of diagnosis, 1024 (76.65%) of all UMs were localized in the choroid; 151 (11.30%) in the choroid and ciliary body; 49 (3.67%) in the iris; 71 (5.31%) in the iris and ciliary body; 29 (2.17%) in the ciliary body; and 12 (0.90%) in the iris, ciliary body, and choroid. In men, tumors tended to be larger than in women, but this difference was not clinically significant (T4 tumors were found in 18.03% men vs. 14.46% women, *p* = 0.068) (Table 3). In detail, 347 (25.97%) of all UMs were classified as T1, 392 (29.35%) as T2, 382 (28.59%) as T3, and 215 (16.09%) as T4.

**Figure 2:**
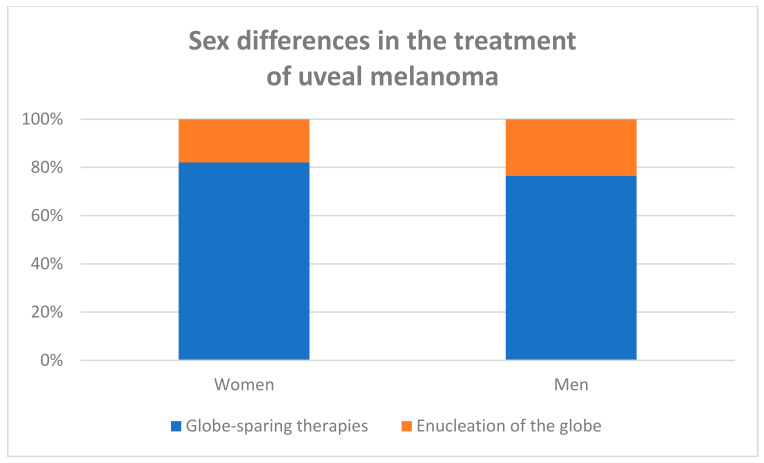
The treatment methods of uveal melanoma according to sex of patient. Chi^2 Pearson test *p* = 0.015.

**Figure 3:**
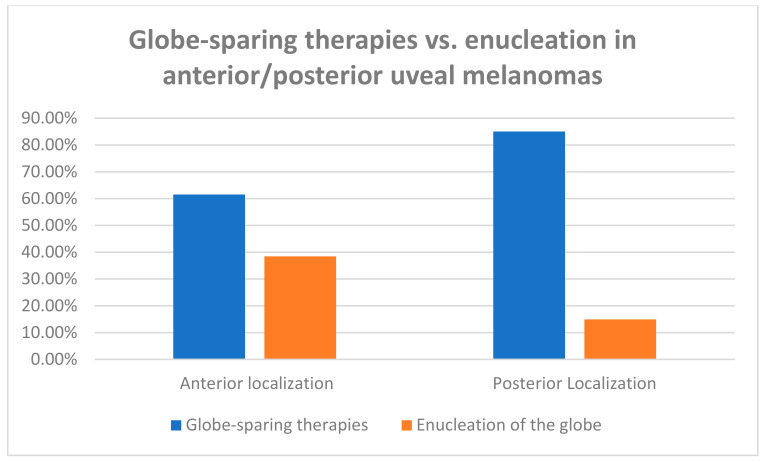
The treatment methods of uveal melanoma according to intraocular anterior/posterior localization of the tumor at the time of cancer diagnosis. Chi^2 Pearson test *p* < 0.001.

Women were treated with globe-sparing therapies (plaque radiotherapy, PBI, and local surgery) more often than men (81.96% vs. 76.56%); contrastingly, men were enucleated more often than women (23.44% vs. 18.04%) (Chi^2 Pearson test *p* = 0.015). The analyses of the medical management of our patients are presented in Table 4, and Figure 2 and Figure 3. The detailed analysis of treatment methods, including plaque brachytherapy with iodine-125 or rhutenium-106, proton beam irradiation (PBI), local surgery combined with plaque brachytherapy, and enucleation of the eye globe, confirmed the sex differences (*p* = 0.014). In total, 909 (68.03%) of all tumors were treated with plaque brachytherapy: 405 (30.31%) with iodine-125 and 504 (37.72%) with rhutenium-106; 36 (2.70%) of tumors were treated with local surgery combined with plaque brachytherapy (either with iodine-125 or rhutenium-106), and 117 (8.76%) were treated with proton beam irradiation (PBI). Enucleation as a primary treatment was provided in 274 (20.51%) of tumors. Additionally, tumors located anterior to the equator were enucleated more often than posterior UMs, and this finding was also statistically significant (38.46% vs. 14.94%, Chi^2 Pearson test *p* < 0.001). Univariate and multivariate logistic regression models are presented in Table 5. All univariate logistic regression models showed statistically significant association between enucleation and gender (women vs. men) (OR 0.72, 95% CI 0.55–0.96, *p* = 0.024), anterior localization of the melanoma (to the equator of the eye globe) (OR 3.56, 95% CI 2.67–4.73, *p* < 0.000), and a larger tumor size, according to the TNM classification (OR 3.46, 95% CI 2.92–4.09, *p* < 0.000). The multivariate logistic regression model showed a statistically significant association between enucleation and anterior localization of the melanoma (to the equator of the eye globe) (OR 4.18, 95% CI 2.96–5.89, *p* < 0.000); a larger tumor size, according to the TNM classification (OR 3.48, 95% CI 2.92–4.15, *p* < 0.000); and that tumors tended to be less frequently enucleated in women (OR 0.73, 95% CI 0.59–1.02, *p* = 0.063).

## 4. Discussion

To the best of our knowledge, this is the first study of uveal melanoma showing the sex differences in the treatment of this malignant tumor. In total, 1336 patients were diagnosed with uveal melanoma and treated between 2018 and 2021 in the national referral center (Department of Ophthalmology and Ophthalmic Oncology, Jagiellonian University Collegium Medicum, Krakow, Poland), and the majority were women (54.34% of patients). According to data from the National Cancer Registry (NCR) of Poland, our sample was a fair representation of the uveal melanoma population in Poland in terms of sex distribution (statistical analysis—Chi squared test: *χ2* = 0.20, *p* = 0.6570) [13]. NCR data also revealed that the incidence rate of uveal melanoma in the whole population of Poland is 6.67/1,000,000 person-years, with the majority of patients with ocular melanoma in Poland being women (53.57%) [13]. In other large, demographic-relevant studies such as the SEER-based reports by Singh et al., Bishop et al., and Mahendraraj et al., the age-adjusted incidence rate of ocular melanoma was significantly higher in men [23,24,25]. In Australia, the difference in the incidence of ocular melanoma between sexes was largely due to significantly higher rates in men aged 65 years and older. There were no significant differences in incidence rates between men and women younger than 65 years [26].

In our study population, uveal melanomas were localized statistically significantly more frequently posterior to the equator of the eye globe in men than in women (79.67% vs. 74.10%). This finding was similar to previously published studies from the British Isles and Israel [17,18], which were focused on gender differences in the clinical presentation of uveal melanoma. In the British Isles, in a population from The Liverpool Ocular Oncology Centre (LOOC), tumors in men were more likely to originate in the choroid than in women (91.7% vs. 87.2%). In the Israelian national referral center (Hadassah-Hebrew University Medical Center, Jerusalem), the tumors located posterior to the equator were found in 86.15% of male and 82.66% of female patents.

However, although we did not compare the exact dimensions of uveal melanomas, tumors tended to be larger in men than in women (T4 tumors were found in 18.03% men vs. 14.46% women). In the aforementioned study from LOOC, Damato and Coupland found that uveal melanomas were larger in men, and this finding was statistically significant [17]. Our results were in agreement with the study from the Israelian national referral center, where significant gender differences were not found for tumor size [18].

We found statistically significant differences between women and men in the treatment of uveal melanoma, i.e., women were treated with globe-sparing therapies (plaque radiotherapy, PBI, local surgery) more often than men (81.96% vs. 76.56%); contrastingly, men were enucleated more often than women (23.44% vs. 18.04%). As all patients were counselled about the benefits of different treatment methods for their survival by the same surgeon (B R-D), we suspect that women were less eager for enucleation due to their greater fear of the cosmetic defects it causes. The previously published analysis from the United States of America (USA) revealed that patients who were not from a White background and were socioeconomically disadvantaged were more likely to receive primary treatment by enucleation, but no interactions were observed between race/ethnicity, socioeconomic status, and tumor stage at the time of diagnosis. This study also revealed that treatment with primary enucleation with no race/ethnic or socioeconomic status statistically significantly decreased the five-year survival rate [27]. In Israel, although men suffered more metastases and a higher mortality rate than women, the sex differences in the treatment modalities were not statistically significant [18]. In the previously published analysis from National Cancer Registry of Poland, the risk of death within five years from the initial ocular melanoma diagnosis was increased by male sex by 1.29 [13]. In our study, men were enucleated more often than women, but we did not study the survival rate (our short period of observation was affected by the COVID-19 pandemic, with possible bias of the cause of death in many of the patients). We believe that the lack of survival analysis likely had only a minor impact on the study findings; the large population size is the major strength of the present study.

In summary, the introduction of globe-sparing therapies in recent years did not decrease the overall survival rate in patients with uveal melanoma [25,28,29]. However, in our study group, tumors were located more anteriorly in women (these tumors are generally enucleated more often), and the number of enucleations in women was statistically significantly lower than in men, probably due to possible fears of cometic defects. As our study population reflects the overall uveal melanoma population in Poland, we suspect that the genetic and hormonal differences between the sexes had the largest impact on clinical presentation and prognosis for those patients [13,19,21]. However, our results are specific to Poland and cannot describe other healthcare systems.

## 5. Conclusions

In this study, statistically significant sex differences were found in the treatment of uveal melanoma in the national referral center in Poland, with men being enucleated more often than women. To the best of our knowledge, this is the first study searching for sex differences in the treatment of uveal melanoma.

## Figures and Tables

**Table 1:** The incidence of uveal melanoma in the national referral center in Poland between 2019 and 2021.

	2018	2019	2020	2021	All
Men n%	171 (46.98%)	157 (43.13%)	132 (45.21%)	150 (47.47%)	610 (45.66%)
Women n%	193 (53.02%)	207 (56.87%)	160 (54.79%)	166 (52.53%)	726 (54.34%)
All n%	364 (100%)	364 (100%)	292 (100%)	316 (100%)	1336 (100%)

**Table 2:** Localizations of uveal melanoma according to sex of patient.

Localization of Tumor	Sex		All n (%)
	Men n (%)	Women n (%)	
Choroid	486 (79.67%)	538 (74.10%)	1024 (76.65%)
Choroid and ciliary body	69 (11.31%	82 (11.30%)	151 (11.30%)
Iris	20 (3.28)	29 (3.99%)	49 (3.67%)
Iris and ciliary body	24 (3.93%)	47 (6.48%)	71 (5.31%)
Ciliary body	7 (1.15%)	22 (3.03%)	29 (2.17%)
Iris, ciliary body and choroid	4 (0.66%)	8 (1.10%)	12 (0.90%)
All	610 (100%)	726 (100%)	1336 (100%)

Chi^2 Pearson test *p* = 0.035.

**Table 3:** Sex distribution in cancer stage according to TNM classification of malignant tumors (at the time of diagnosis).

Cancer Stage TNM	Sex		All n (%)
	Men n (%)	Women n (%)	
T1	170 (27.87%)	177 (24.38%)	347 (25.97%)
T2	165 (27.05%)	227 (31.27%)	392 (29.35%)
T3	165 (27.05)	217 (29.89%)	382 (28.59%)
T4	110 (18.03%)	105 (14.46%)	215 (16.09%)
All	610 (100%)	726 (100%)	1336 (100%)

Chi^2 Pearson test *p* = 0.068.

**Table 4:** The treatment methods of uveal melanoma according to sex of patient.

Treatment Methods	Sex		All n (%)
	Men n (%)	Women n (%)	
Plaque brachytherapy with iodine-125	188 (30.82%	217 (29.89%)	405 (30.31%)
Plaque brachytherapy with rhutenium-106	212 (34.75%)	292 (40.22%)	504 (37.72%)
Local surgery with plaque brachytherapy	10 (1.65)	26(3.58%)	36 (2.70%)
Proton beam irradiation (PBI)	57 (9.34%)	60 (8.27%)	117 (8.76%)
Enucleation	143 (23.44%)	131 (18.04%)	274 (20.51%)
All	610 (100%)	726 (100%)	1336 (100%)

Chi^2 Pearson test *p* = 0.014.

**Table 5:** Univariate and multivariate logistic regression models for risk factors associated with medical management of uveal melanoma (globe-sparing therapies vs. enucleation).

Variables	Univariate Logistic Regression OR, 95% CI, *p*-Value	Multivariate Logistic Regression OR, 95% CI, *p*-Value
Gender: women vs. men	0.72 (0.55–0.96) ***p* = 0.024**	0.73 (0.59–1.02) *p* = 0.063
Tumor localization: anterior vs. posterior to the equator of eye globe Size: according to TNM Classification	3.56 (2.67–4.73) ***p* < 0.000** 3.46 (2.92–4.09) ***p* < 0.000**	4.18 (2.96–5.89) ***p* < 0.000** 3.48 (2.92–4.15) ***p* < 0.000**

Bold highlights the statistically significant *p* values.

## Data Availability

Not applicable.
